# Gender and Racial Disparities in Diagnostic Radiology Residency Programs: A Decade of Trends and Implications for Diversity and Inclusion

**DOI:** 10.7759/cureus.91837

**Published:** 2025-09-08

**Authors:** Imran Bitar, Mazen Zamzam, Hassan Alzayadi, Kamil Abushaban, Daniel Piziali

**Affiliations:** 1 School of Medicine, Oakland University William Beaumont School of Medicine, Rochester, USA; 2 Department of Biology, University of Michigan, Dearborn, USA; 3 Department of Diagnostic Radiology and Molecular Imaging, Corewell Health William Beaumont University Hospital, Royal Oak, USA

**Keywords:** accreditation council graduate medical education, equity diversity and inclusion, radiology, residency gender diversity, underrepresented

## Abstract

As the United States moves toward becoming a minority-majority nation by 2060, achieving diversity within the physician workforce has become increasingly important. Diagnostic Radiology, however, continues to demonstrate persistent disparities in representation, particularly among racial, ethnic, and gender minorities. This study examined gender, racial, and ethnic representation among Diagnostic Radiology residents from 2013 to 2023 using data from the Accreditation Council for Graduate Medical Education Data Resource Book. Residents self-reported their race and ethnicity into defined categories. Data trends were analyzed over the decade to assess shifts in representation. Of 46,557 Diagnostic Radiology residents, 33,871 (72.8%) were male and 12,431 (26.7%) were female. Racial and ethnic breakdowns included 24,482 (52.6%) White, 10,297 (22.1%) Asian, 2,126 (4.57%) Hispanic/Latino, 1,561 (3.35%) Black/African American, 83 (0.18%) American Indian/Alaskan Native, 10 (0.021%) Native Hawaiian/Pacific Islander, 615 (1.32%) multiracial, 2,246 (4.82%) other, and 5,137 (11%) unknown. Overall, 47.4% of residents identified as non-White. Despite increasing attention to diversity and inclusion, Diagnostic Radiology remains one of the least diverse medical specialties. Contributing factors include limited exposure to radiology during medical school, lack of mentorship, and structural barriers in the residency selection process. Targeted efforts such as diversity, equity, and inclusion (DEI) committees, pipeline programs, and holistic applicant reviews have emerged as key strategies to promote diversity.

## Introduction

The US Census Bureau projects that by the year 2060, the racial and ethnic makeup of the US population will become a minority-majority country. It is projected that 56.4% of all Americans will belong to a minority group by 2060 [1], as compared to 38.4% of Americans in 2020 [2]. Yet, despite these shifts, the field of Diagnostic Radiology has shown limited advancement in achieving meaningful diversity, equity, and inclusion (DEI), particularly for underrepresented racial and ethnic minorities [3].

Several studies have documented persistent disparities among Radiology trainees. A national analysis found that women and underrepresented in medicine (URM) groups were significantly underrepresented in Diagnostic Radiology compared to their proportions in the general population and among medical school graduates [3]. More recent studies confirm that these disparities remain largely unchanged, with only modest gains in URM representation across Radiology residencies from 2013 to 2019 [4] and underrepresentation continuing among Black, Hispanic, and Native American applicants [5].

Gender disparities are similarly well-documented. While women now comprise over 50% of US medical school matriculants, they remain significantly underrepresented in Diagnostic Radiology training programs [6]. Studies examining recent match data from 2022 to 2024 highlight a stagnation in female representation, despite institutional efforts to increase recruitment through mentorship and outreach [7].

The racial and ethnic compositions of primary care physicians have also been studied, further revealing the underrepresentation of minority groups [8]. This underrepresentation extends to graduate medical education (GME) programs, where disparities in race, ethnicity, and gender persist across multiple specialties, including Diagnostic Radiology [9]. This study aims to evaluate gender, racial, and ethnic representation among Diagnostic Radiology residents from 2013 to 2023 using Accreditation Council for Graduate Medical Education (ACGME) data, with the goal of identifying disparities and informing future diversity initiatives.

## Materials and methods

Data on Diagnostic Radiology residents from 2013 to 2023 were gathered and analyzed to identify trends in gender and ethnic representation. These data were sourced from the ACGME Data Resource Book [10], which provides publicly available information and, therefore, does not require institutional review board (IRB) approval. 

All Diagnostic Radiology residents recorded in the ACGME Data Resource Book for the years 2013-2023 were included. No residents were excluded from analysis. Resident counts were extracted directly from ACGME annual summary tables, which present aggregated demographic data. Data for gender, race, and ethnicity were manually extracted from the annual tables in the ACGME Data Resource Book. Categories were recorded exactly as reported by the ACGME: White, Asian, Black/African American, Hispanic/Latino, American Indian/Alaskan Native, Native Hawaiian/Pacific Islander, multiracial, other, and unknown. Gender categories included male, female, non-binary/other, and not reported. Data for Native Hawaiian/Pacific Islander were unavailable from 2013 to 2019, and for multiracial, data were unavailable from 2013 to 2020. When categories were not reported in earlier years, they were treated as missing rather than imputed or pooled. "Unknown" and "Other" categories were retained as independent groups to avoid bias introduced by exclusion or reassignment. Descriptive statistics (counts and percentages) were calculated for each gender and racial/ethnic category by year for the entire decade. Each category was analyzed separately; no categories were pooled or excluded. Trends over time were evaluated descriptively and are presented graphically.

## Results

The total number of Diagnostic Radiology residents in the decade-long analysis was 46,557. Of this total number, 33,871 residents (72.8%) were male, 12,431 (26.7%) were female, 254 (0.55%) were not reported, and one was non-binary.

Self-identified races and ethnicities of residents were evaluated, and each group made up the following portions: 24,482 Whites (52.6%); 10,297 Asians (22.1%); 2,126 Hispanic/Latino (4.57%); 1,561 Black/African American (3.35%); 83 American Indian or Alaskan Native (0.18%); 10 Native Hawaiian or Pacific Islander (0.021%); 615 multiple race/ethnicity (1.32%); 2,246 other (4.82%); and 5,137 unknown (11%). The total number of residents identifying as White was 24,482 (52.6%), while 22,075 residents were identified as non-White (47.4%). For the years 2013-2019, data for the Native Hawaiian or Pacific Islander group were not available. For the years 2013-2020, data for the multiple race/ethnicity group were not available.

**Figure 1:**
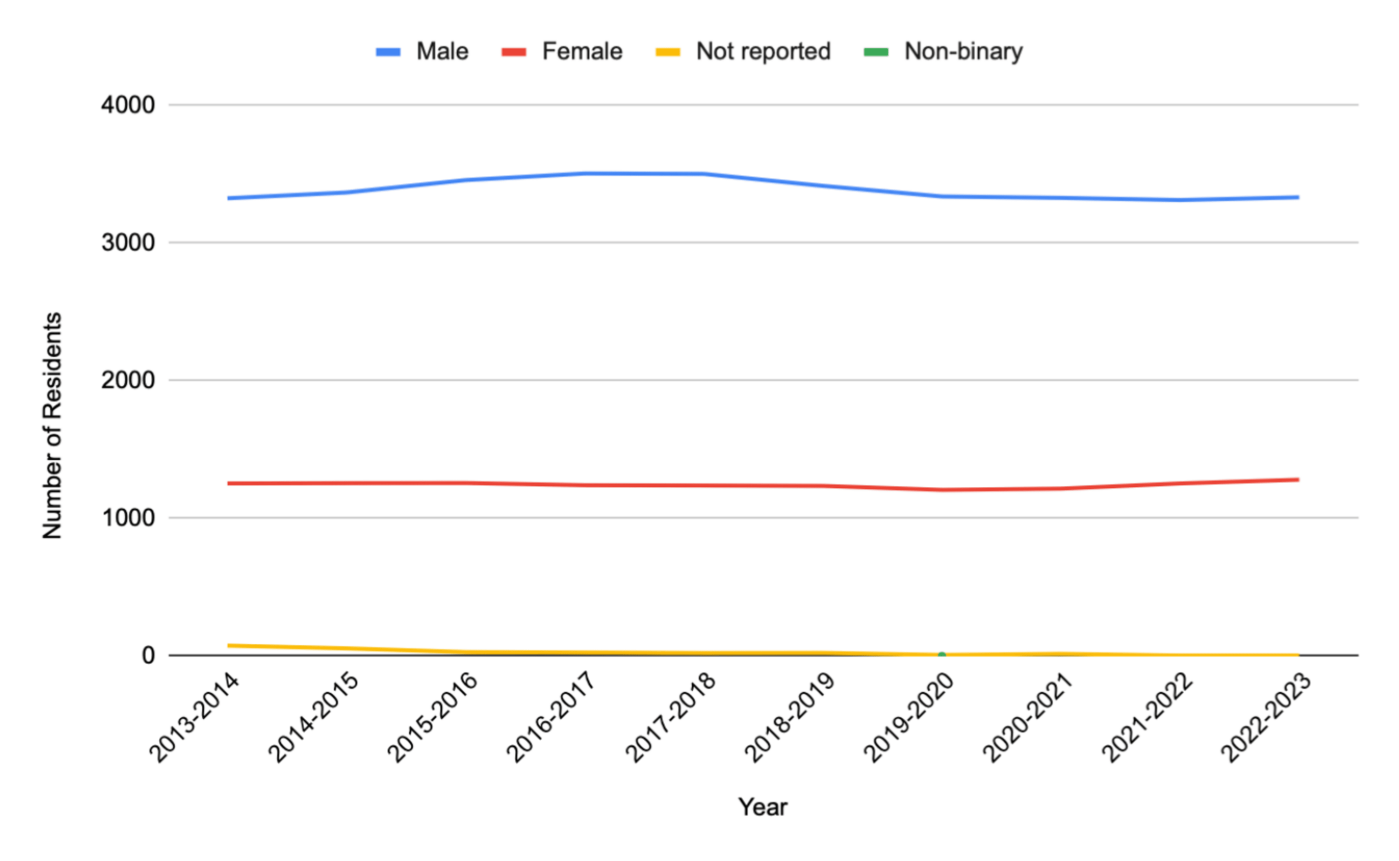
In the years 2013-2023, the yearly change of Diagnostic Radiology residents by gender

**Figure 2:**
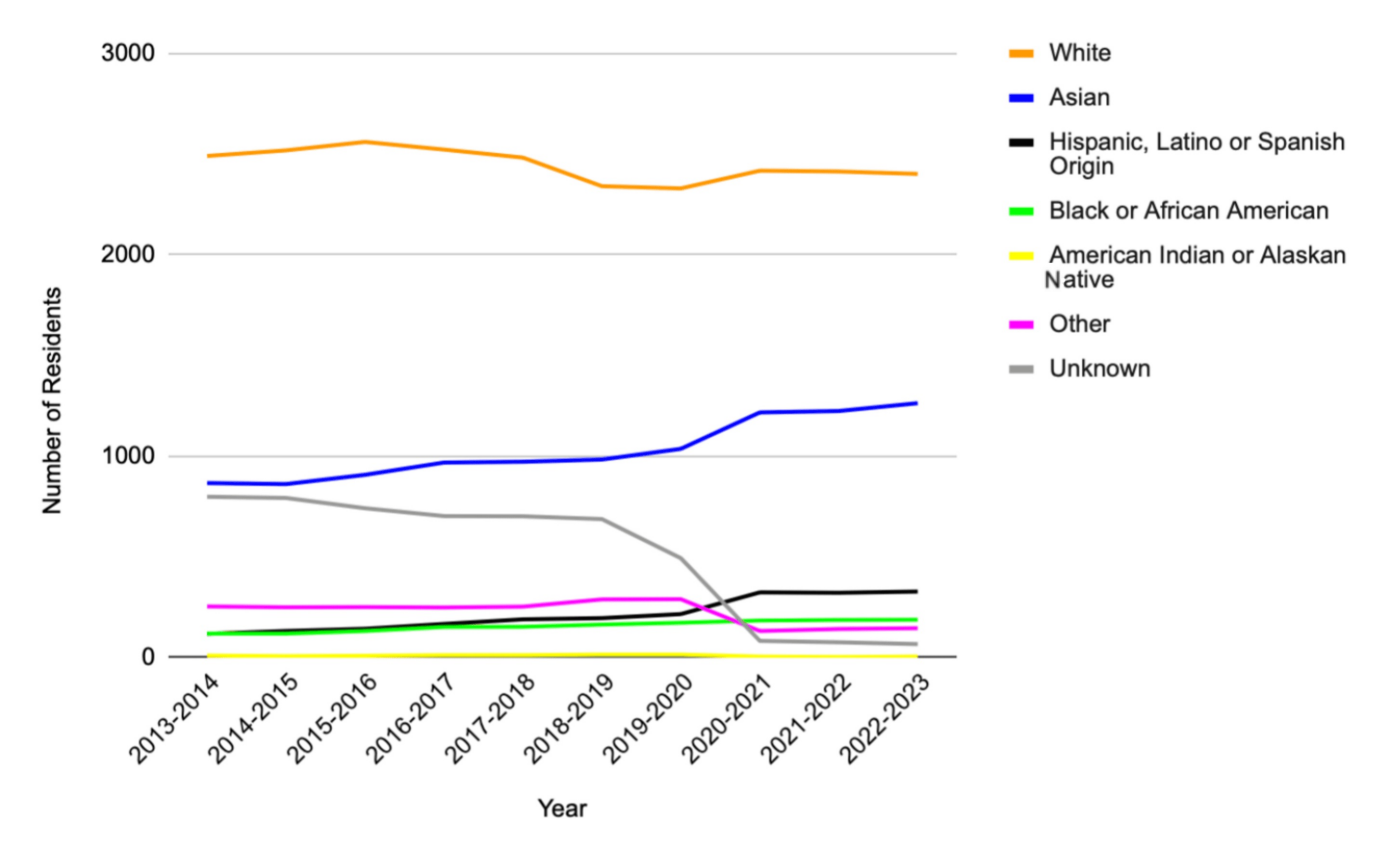
In the years 2013-2023, the yearly change of Diagnostic Radiology residents by racial and ethnic group

**Figure 3:**
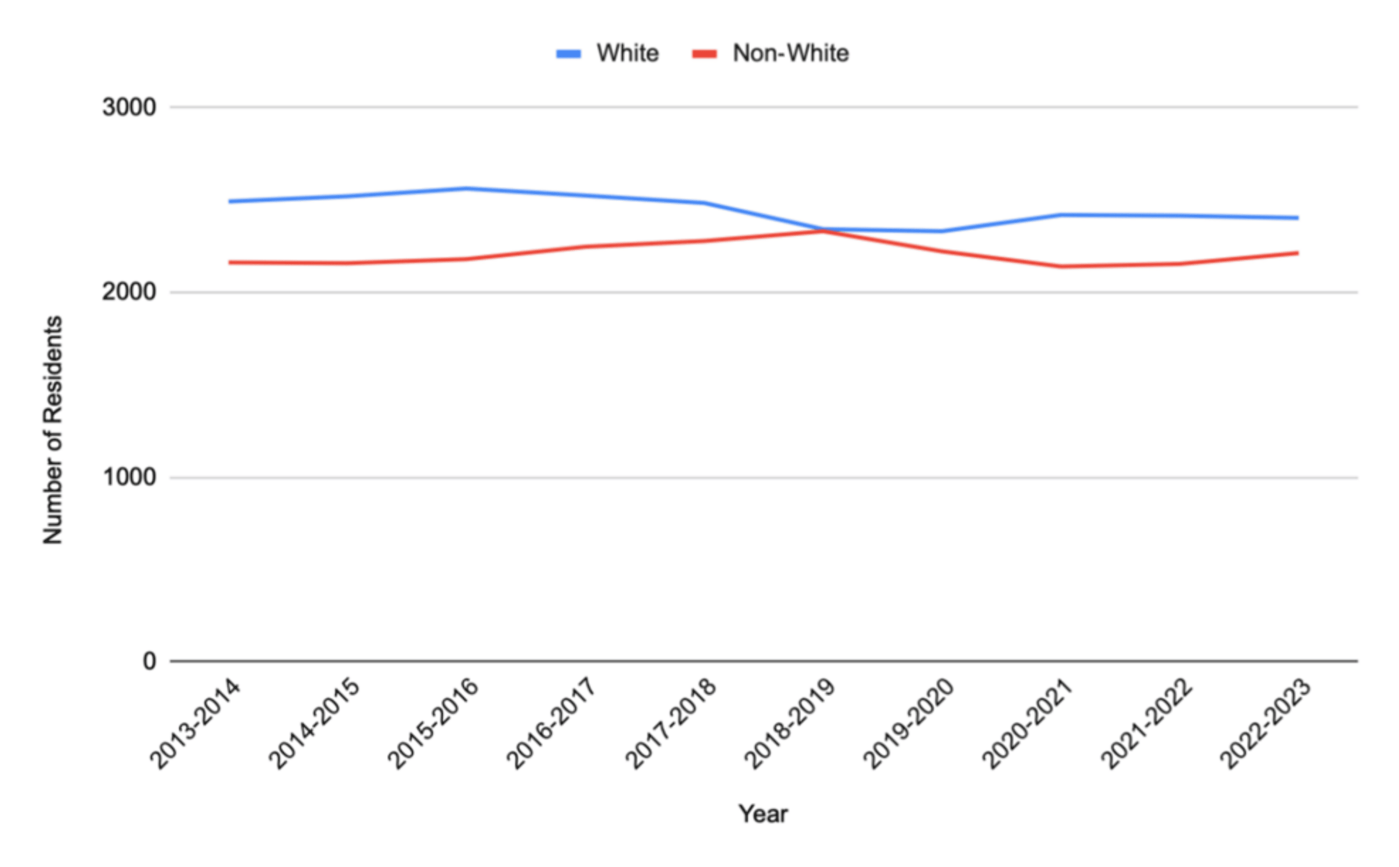
In the years 2013-2023, the number of White and non-White Diagnostic Radiology residents

Table 1 provides an overview of the compiled data of Diagnostic Radiology residents from 2013 to 2023. Figures 1-3 display the gender representation of Diagnostic Radiology residents from 2013 to 2023, the racial and ethnic representation of Diagnostic Radiology residents from 2013 to 2023, and the White and non-White representation of Diagnostic Radiology residents from 2013 to 2023, respectively.

**Table 1:** Number of Diagnostic Radiology residents arranged by race, ethnicity, and sex. Data for the Native Hawaiian/Pacific Islander group were not collected from 2013 to 2019. Data for the multiple race/ethnicity group were not collected from 2013 to 2020

Year	Total residents	Male	Female	Not reported (gender)	Non-binary	White	Asian	Hispanic, Latino, or Spanish origin	Black or African American	American Indian or Alaskan Native	Native Hawaiian/Pacific Islander	Multiple race/ethnicity	Other (race)	Unknown (race)
2013-2014	4652	3324	1253	75	-	2491	866	117	118	9	-	-	253	798
2014-2015	4676	3366	1255	55	-	2519	861	131	118	6	-	-	249	792
2015-2016	4740	3456	1256	28	-	2561	907	142	131	8	-	-	250	741
2016-2017	4769	3504	1240	25	-	2523	968	166	151	11	-	-	248	702
2017-2018	4760	3501	1238	21	-	2483	972	189	152	11	-	-	252	701
2018-2019	4671	3414	1235	22	-	2341	983	195	163	14	-	-	288	687
2019-2020	4551	3337	1206	7	1	2330	1036	215	172	14	2	-	289	493
2020-2021	4557	3327	1215	15	-	2418	1217	323	183	4	3	196	131	82
2021-2022	4567	3311	1253	3	-	2414	1224	321	186	2	3	201	141	75
2022-2023	4614	3331	1280	3	-	2402	1263	327	187	4	2	218	145	66
Total	46557	33871	12431	254	1	24482	10297	2126	1561	83	10	615	2246	5137

## Discussion

The analysis of Diagnostic Radiology residents from 2013 to 2023 reveals notable gender and racial disparities within the field. Of the residents analyzed, 72.8% were male and 26.7% were female, indicating a persistent gender imbalance. This gender disparity aligns with previous research, which has consistently shown that Diagnostic Radiology has struggled to recruit and retain diverse residents compared to other medical specialties [3].

Significant racial and ethnic disparities were also observed. These findings mirror patterns reported in our prior work examining Pediatric Orthopaedic Surgery fellows, where similar underrepresentation was evident despite increased attention to diversity and inclusion across medical education [11]. Previous studies suggest that systemic barriers, including a lack of mentorship, limited exposure to Radiology during medical school, and implicit biases in the residency selection process, contribute to disparities in recruitment and retention [12]. Spottswood et al. found that historically, URMs faced structural disadvantages in accessing competitive medical specialties, including Diagnostic Radiology [13]. Addressing these barriers requires a multifaceted efforts that expand access and promote inclusion.

Targeted initiatives have been implemented to improve representation, including structured mentorship programs, leadership development, and inclusion-focused recruitment strategies [12]. For example, institutions have created DEI committees to guide policy changes and advocate for systemic improvements within Radiology departments [14]. These committees play a crucial role in identifying barriers to diversity and implementing actionable solutions to improve representation.

Women in Radiology also face unique challenges regarding career advancement and representation. The impact of gender-focused initiatives, such as the Women in Radiology Program, has been instrumental in fostering an inclusive environment that supports female radiologists in achieving leadership positions and professional growth [15]. In addition to gender diversity, increasing the number of radiologists from underrepresented racial and ethnic backgrounds is essential for addressing healthcare disparities, particularly in underserved populations [12]. Studies indicate that medical school graduates who come from diverse backgrounds are more likely to serve in underserved communities, emphasizing the importance of diversity initiatives in Radiology training programs [16].

A review of the literature suggests that effective strategies to improve diversity in Diagnostic Radiology and other medical specialties combine early pipeline development, holistic review in residency selection, and structured mentorship. Initiatives that target undergraduate and medical students, particularly those from URM backgrounds, have been shown to increase application rates to competitive specialties, including Radiology [13]. Furthermore, holistic review practices that integrate mission alignment, applicant experiences and attributes, and active bias mitigation have been shown to enhance diversity in residency recruitment [17]. Boatright et al. outlined a comprehensive set of strategies, ranging from community engagement and holistic admissions review to implicit bias training, inclusive selection committees, and longitudinal DEI curricula, that provide residency programs with practical tools to integrate DEI throughout the stages of medical training [18]. Together, these evidence-based approaches offer guidance for residency programs seeking to address the persistent challenges associated with diversity gaps in Diagnostic Radiology.

While this study provides a decade-long overview of gender, racial, and ethnic representation in Diagnostic Radiology residency programs, certain limitations should be considered when interpreting the findings. The "Unknown" category for race/ethnicity (11%) reduces the accuracy of representation estimates. If underrepresented minorities were disproportionately classified in the "Unknown" group, this may bias estimates downward. Additionally, missing data for certain categories in earlier years (such as Native Hawaiian/Pacific Islander, multiracial) may lead to the underestimation of representation in these groups. This also limits comparability across years, as these categories were not consistently reported throughout the dataset. Small subgroup counts (e.g., Native Hawaiian, American Indian) should be interpreted with caution, as percentages may exaggerate changes and may overstate year-to-year changes. Moreover, because our analysis was descriptive, the robustness of the findings is limited to observed counts and percentages rather than statistical associations. Finally, this study focuses solely on representation and does not assess outcomes beyond residency entry, such as retention, career advancement, or leadership attainment.

## Conclusions

This study highlights the persistent gender and racial disparities within Diagnostic Radiology residency programs from 2013 to 2023. Despite ongoing diversity and inclusion efforts, women and underrepresented minority groups remain significantly underrepresented in the field. As this analysis was descriptive and did not include statistical testing, observed trends should be interpreted cautiously, as they may reflect either meaningful change or random variation. Addressing these disparities requires a multifaceted approach, including mentorship programs, holistic selection processes, and institutional initiatives aimed at fostering a more inclusive environment. Increasing representation in Diagnostic Radiology may play an important role in promoting equitable healthcare delivery and addressing disparities in patient outcomes, particularly in underserved populations.
